# Investigating Skin Rashes Associated With Pantoprazole Medication: Causes and Clinical Implications

**DOI:** 10.7759/cureus.44623

**Published:** 2023-09-03

**Authors:** Suhasini Krishnan, Siddharth Sankar Das, Sanjana Mahanta, Susmita Das, Ena Skikic

**Affiliations:** 1 Department of Medical Education, Dubai Academic Health Corporation, Dubai, ARE; 2 Department of General Surgery, Dubai Hospital, Dubai, ARE; 3 Department of Obstetrics and Gynecology, Aster DM Hospital, Dubai, ARE

**Keywords:** hypersensitivity, skin rashes, drug reaction, adverse, pantoprazole

## Abstract

Pantoprazole is a proton-pump inhibitor mainly used in treating various gastroesophageal disorders and frequently as prophylaxis for stress ulcers and gastrointestinal bleeding in most patients admitted for in-hospital management. Hypersensitivity reactions to this medication have been reported, although the exact incidence and prevalence are unknown. Further studies on proton-pump inhibitor allergic reactions should be conducted to enable physicians to safely select and prescribe an alternative type of medication within the same drug class, confidently avoiding the allergenic molecular compound that the patient reacted to previously. We present a case of a 35-year-old male postoperative bariatric patient with no significant allergy history who developed an allergic skin rash a week after being discharged on pantoprazole 40 mg. His rash was itchy and distributed mainly over the torso and lower limbs, without any additional respiratory or gastrointestinal symptoms.

## Introduction

Pantoprazole belongs to a class of medications known as proton-pump inhibitors (PPIs), which are most commonly used in the treatment of gastroesophageal disorders such as gastroesophageal reflux disease (GERD), *Helicobacter pylori* infections, and prophylaxis in peptic ulcer disease (PUD) to prevent bleeding, in chronically ill patients, or in those undergoing bariatric surgeries. Its mechanism of action is irreversible inhibition of the hydrogen-potassium adenosine triphosphate (H+/K+ ATP) enzyme pumps, which are commonly found in the parietal cells of the stomach lining. This inhibition results in decreased gastric acid secretion for up to 24 hours. It may be safely prescribed for both adult and pediatric patients. Its adverse effects include, but are not limited to, abdominal pain, diarrhea, electrolyte imbalances, and vitamin deficiencies. The drug is contraindicated in those patients who have shown hypersensitivity previously, which can present with angioedema, urticaria, bronchospasm, and anaphylactic shock [1]. It has been theorized that the modifications of the side chains in the chemical structure of the drugs, which serve as differentiation between them, are the main contributors to the cross-reaction and subsequent hypersensitivity development [2,3]. 

Patients undergoing bariatric procedures risk developing marginal or anastomotic ulcers due to acid secretion, which can cause bleeding and increase morbidity. Therefore, it would be prudent to prescribe a proton-pump inhibitor to these patients, although there are variations in existing literature regarding the recommended duration of postoperative PPI administration [4].

## Case presentation

A 35-year-old obese male with a pre-operative weight of 132 kg, height of 172 cm, and body mass index (BMI) of 44.6 kg/m2 was admitted to the hospital for laparoscopic sleeve gastrectomy. His comorbidities include obstructive sleep apnea and hypertension. He has been on medication for two years. He underwent a successful laparoscopic sleeve gastrectomy. Postoperatively, the patient recovered well, was discharged from the hospital in stable condition, and was counseled on adequate oral intake. As per standard post-sleeve gastrectomy protocol, the patient was prescribed oral multivitamins, calcium effervescent tablets of 600 mg, vitamin D tablets of 50000 IU weekly, and pantoprazole 40 mg daily. 

**Figure 1:**
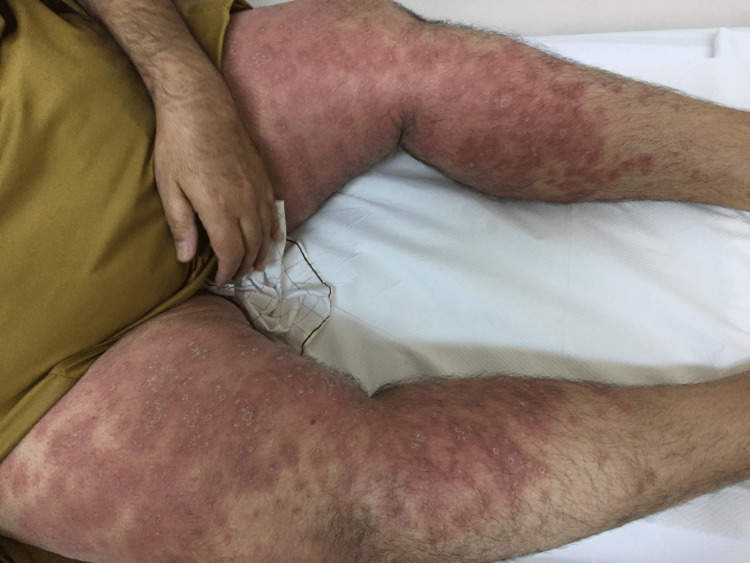
Erythematous maculopapular eruption with some excoriations along the anteromedial aspect of the lower limbs.

**Figure 2:**
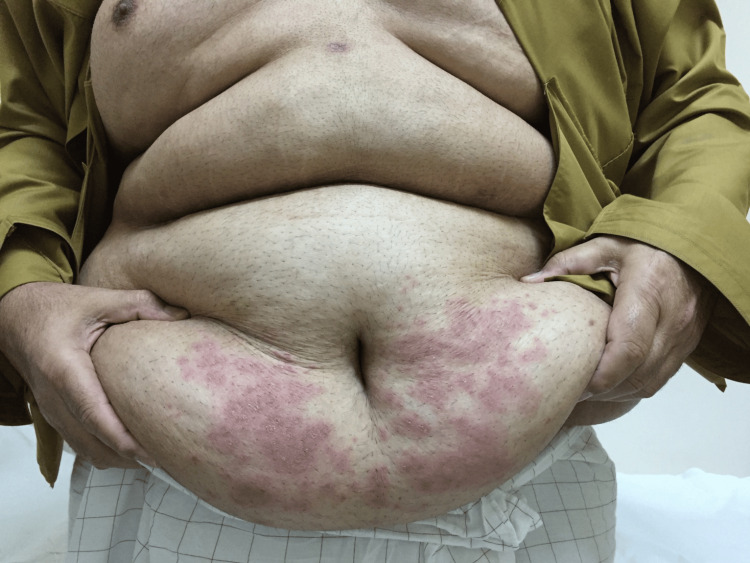
Erythematous maculopapular eruption in the periumbilical region.

**Figure 3:**
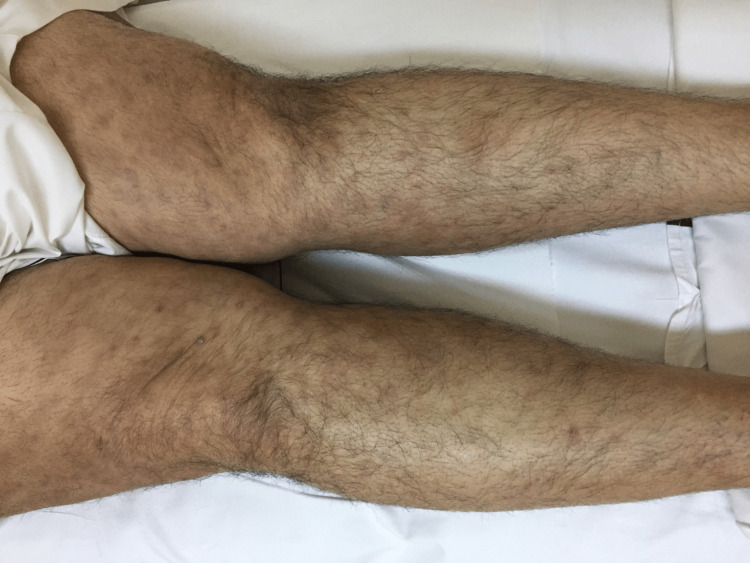
Resolution of eruption along the anteromedial aspect of the lower limbs with some residual post-inflammatory hyperpigmentation.

**Figure 4:**
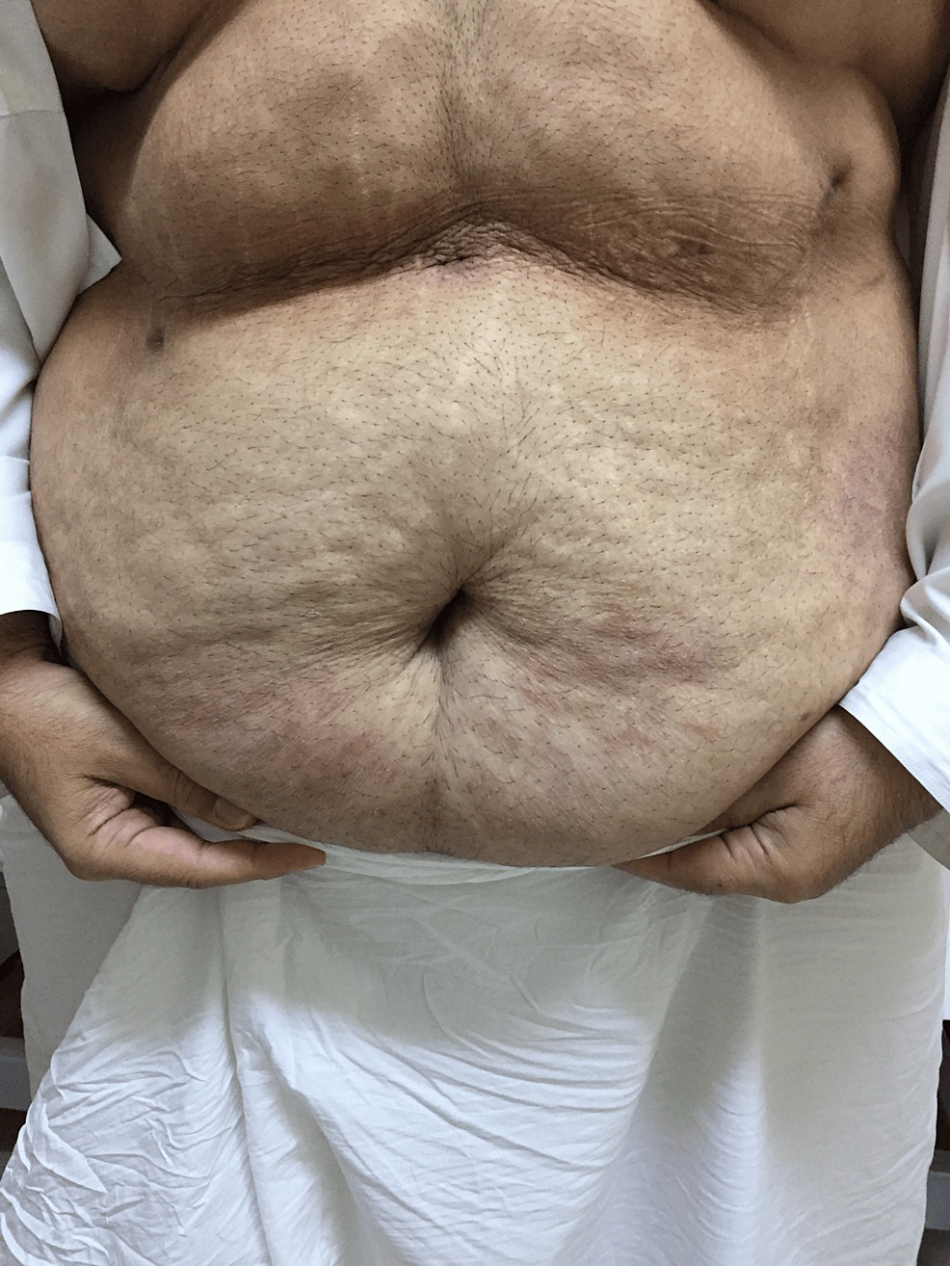
Resolution of the eruption in the periumbilical region with some residual post-inflammatory hyperpigmentation.

The patient was also advised to follow up at the bariatric clinic after two weeks. He defaulted to follow-up and revisited the clinic after one month of surgery. Following surgery, his weight was reduced to 118 kg, with a new BMI of 39.9 kg/m2. His oral intake was reported to be around 1500 ml to 1800 ml every 24 hours. He presented to the clinic with complaints of new-onset itchiness and rashes that developed all over his body, primarily in the lower limbs, abdomen, chest, and back. He denied any symptoms of difficulty breathing or wheezing, swelling of the face, difficulty swallowing, nausea or vomiting, changes in bowel movements, urinary complaints, or fever. On inspection, a diffuse erythematous and edematous maculopapular rash was noted along both upper limbs, lower limbs, and the periumbilical region, as evidenced in Figures 1, 2. Mucous membranes were unaffected. There was no facial angioedema noted. The patient was admitted to the hospital for observation for two days. All laboratory investigations were unremarkable. Pantoprazole was stopped, and the patient was started on a levocetirizine hydrochloride tablet of 5 mg, following which he reported good improvement in symptoms. He was then discharged with advice to follow up in the clinic after one week. Upon follow-up, nearly 70% of the rash had disappeared, and after two weeks, he reported a complete resolution of symptoms. Improvement of the rash can be seen in Figures 3, 4. He was advised to avoid proton-pump inhibitor intake in the future.

## Discussion

PPIs are chemically structured as modified benzimidazoles with a pyridine ring. They may be differentiated on the basis of the substituents found on the base ring [5]. For example, omeprazole has methoxy side chains in the benzimidazole ring, while pantoprazole has difluoromethoxy side chains. However, in lansoprazole and rabeprazole, it is the pyridine rings, rather than the benzimidazole rings, that are modified to contain trifluoromethoxy and methoxypropoxy side chains, respectively. Due to these variations in molecular structure, cross-reactivity leading to hypersensitivity reactions could occur [2,3]. 

PPIs are usually well tolerated, and any side effects patients report, such as gastrointestinal upset or headaches, are short-term and easily overcome [6]. It has additionally been noted that these adverse reactions account for just 0.2% to 0.7% of all cases of anaphylaxis [7]. However, reports of reactions have become increasingly frequent, which could be explained by overprescription or the ease of over-the-counter access [6]. 

Although uncommon, hypersensitivity reactions to PPIs can vary in their presentation, from urticaria, as is seen with our patient, to angioedema and reactions such as drug rash with eosinophilia and systemic symptoms (DRESS) [8,9]. One five-year retrospective study conducted by Casciaro and colleagues reported that all types of PPIs were capable of inducing a hypersensitivity reaction. They highlighted that omeprazole was found to be associated with the highest incidence of such reactions, followed by pantoprazole and lansoprazole [10]. 

One of the earliest reports of angioedema and urticaria after proton-pump inhibitor ingestion was in 1992. A 44-year-old patient reported experiencing the previously mentioned symptoms two hours after omeprazole intake, specifically in capsule form. When subsequently ingesting enteric-coated granules without the capsule shell, no symptoms were reported, suggesting sensitivity to one of the capsule’s components (gelatin, red iron oxide, titanium dioxide, or printing ink) rather than the medication itself [11]. 

A literature review conducted by Lombardo and colleagues found that urticaria or angioedema accounted for 58% of PPI hypersensitivity cases, while 40% manifested as anaphylactic reactions. The authors further emphasized the importance of exercising caution when prescribing the medications to patients who were known to have a *Helicobacter pylori* infection or known hypersensitivity to non-steroidal anti-inflammatory drugs or antibiotics [8]. 

For patients who have experienced severe reactions to PPIs, desensitization to the drug could be a beneficial option. A case report by Confino-Cohen and colleagues described a 44-year-old patient with an active *Helicobacter pylori* infection who, during the third round of triple therapy, developed an anaphylactic reaction to omeprazole. Through successful desensitization, the patient was restarted on omeprazole tablets 20 mg twice daily, which resulted in the complete resolution of symptoms and eradication of the infection. However, this option presents its disadvantages, which include high costs and the complexity of the desensitization process [12]. 

## Conclusions

While proton-pump inhibitor hypersensitivity reactions are increasingly reported, further analyses and investigations are imperative to assessing their true incidence. Understanding the correlation of reactions with the molecular structures and chemical compositions of different drugs within the proton-pump inhibitor drug class would enable physicians to select the most suitable medication for their patients, avoiding cross-reactions. Counseling patients on symptoms and signs of adverse reactions would not only result in providing prompt medical attention but also aid in accurate adverse event reporting and data keeping.
